# Supplementary material for: Cytogenetic, Morphometric, and Ecological Characterization of Festuca indigesta Boiss. in the Southeast of Spain
Source: Plants (Basel). 2022 Mar 4;11(5):693. doi: 10.3390/plants11050693 (PMC8912771; doi:10.3390/plants11050693)
Supplement: Supplementary file 1 [file plants-11-00693-s001.zip › Table S1.pdf]

Table S1. Morphometric data for cytotypes of *Festuca indigesta* subsp. *indigesta*. Different letters represent statistically significant differences at $p < 0.05$.

Character	Cytotype	Mean ± SD	Min	Max
Culm length (cm)	12*x*	33.67 ± 11.17[a]	12.00	50.00
	8*x*	40.59 ± 5.84[b]	25.00	51.00
	6*x*	30.91 ± 3.64[a]	23.50	39.50
Culm diameter (mm)	12*x*	0.66 ± 0.14	0.50	1.00
	8*x*	0.66 ± 0.09	0.50	0.80
	6*x*	0.57 ± 0.08	0.40	0.80
Leaf diameter (mm)	12*x*	0.87 ± 0.12	0.70	1.10
	8*x*	0.91 ± 0.12	0.70	1.30
	6*x*	0.90 ± 0.10	0.70	1.10
Auricle length (mm)	12*x*	0.32 ± 0.12[a]	0.10	0.70
	8*x*	0.52 ± 0.21[b]	0.20	1.00
	6*x*	0.59 ± 0.32[b]	0.10	1.40
Panicle length (cm)	12*x*	5.87 ± 1.98[ab]	3.00	10.00
	8*x*	6.18 ± 1.27[b]	4.00	9.20
	6*x*	4.90 ± 0.98[a]	3.00	8.50
Spikelet length (mm)	12*x*	10.15 ± 1.22[c]	8.20	15.00
	8*x*	9.52 ± 1.14[b]	7.30	12.50
	6*x*	8.39 ± 0.64[a]	7.20	10.00
Lower glume length (mm)	12*x*	3.77 ± 0.53[c]	3.00	5.70
	8*x*	3.38 ± 0.41[b]	2.60	4.40
	6*x*	3.07 ± 0.38[a]	2.50	4.20
Upper glume length (mm)	12*x*	5.31 ± 0.63[c]	4.10	7.00
	8*x*	4.89 ± 0.55[b]	3.80	6.70
	6*x*	4.23 ± 0.37[a]	3.50	5.40
Lemma length (mm)	12*x*	6.41 ± 0.54[c]	5.40	7.60
	8*x*	5.84 ± 0.43[b]	4.80	6.70
	6*x*	5.12 ± 0.33[a]	4.50	6.00
Awn length (mm)	12*x*	1.45 ± 0.54	0.60	2.70
	8*x*	1.30 ± 0.35	0.70	2.00
	6*x*	1.32 ± 0.38	0.70	2.50
Number of flowers per spikelet	12*x*	5.47 ± 1.18[ab]	3.30	8.00
	8*x*	5.94 ± 1.27[b]	4.00	9.00
	6*x*	5.08 ± 0.74[a]	4.00	7.00
Anther length (mm)	12*x*	3.42 ± 0.70[c]	2.40	4.90
	8*x*	2.52 ± 0.42[b]	2.00	3.40
	6*x*	2.81 ± 0.37[a]	2.00	3.70
Stomatal accessory cells (μm)	12*x*	30.76 ± 4.84[c]	17.39	41.53
	8*x*	25.83 ± 3.17[b]	17.94	34.27
	6*x*	22.84 ± 3.34[a]	15.79	35.56
Stomatal guard cells of stomata (μm)	12*x*	38.84 ± 5.93[c]	22.93	58.04
	8*x*	33.92 ± 3.80[b]	25.34	43.35
	6*x*	30.12 ± 3.49[a]	22.67	39.37
