# Supplementary material for: Cytogenetic, Morphometric, and Ecological Characterization of Festuca indigesta Boiss. in the Southeast of Spain
Source: Plants (Basel). 2022 Mar 4;11(5):693. doi: 10.3390/plants11050693 (PMC8912771; doi:10.3390/plants11050693)
Supplement: Supplementary file 1 [file plants-11-00693-s001.zip › Table S2.pdf]

Table S2. Eigenvectors showing correlations of morphological characters with the first two principal components.

Character	PC1	PC2
Panicle length	0.274	-0.270
Spikelet length	0.376	-0.196
Lower glume length	0.346	-0.246
Upper glume length	0.402	-0.198
Lemma length	0.424	-0.125
Anther length	0.318	-0.131
Stomatal accessory cells length	0.318	0.654
Stomatal guard cells length	0.347	0.573
